# Analysis of Essential and Nonessential Elements in Local and Imported Raw and Cooked Rice (*Oryza sativa*) Samples in Ethiopia

**DOI:** 10.1155/ianc/1237306

**Published:** 2026-04-28

**Authors:** Tamene Tadesse Beyene, Tesfaye Gudina, Abebe Diro, Mulugeta Tesema Efa, Faisal Mukhtar

**Affiliations:** ^1^ Department of Chemistry, College of Natural Sciences, Jimma University, Jimma, Oromia, Ethiopia, ju.edu.et; ^2^ Department of Chemistry, Collage of Natural & Computational Science, Dambi Dollo University, Dambi Dollo, Oromia, Ethiopia, dadu.edu.et; ^3^ Institute of Physics, The Islamia University of Bahawalpur, Bahawalpur, 63100, Pakistan, iub.edu.pk

**Keywords:** cooked rice, FAAS, heavy metals, metal analysis, rice, wet digestion

## Abstract

This study aimed to measure the concentrations of essential and nonessential metals in raw and cooked rice samples from Ethiopia. Researchers collected rice samples, both local and imported, from the Jimma town market. The samples were stored in prerinsed plastic bags and rinsed with 2 mol/L HNO_3_ and deionized water to prevent contamination. Preparation involved wet digestion methods, and the samples were stored at 4°C until analysis. Flame atomic absorption spectrometry (FAAS) was used to analyze the samples in triplicate, and the results were validated for accuracy, precision, instrument detection limit (IDL), limit of detection (LOD), and limit of quantification (LOQ). The findings revealed significant differences (*p* < 0.05) in the mean concentrations of metals across all rice samples. The metals detected included both essential and nonessential types: chromium (Cr) ranged from nondetectable (ND) to 15.36 mg/kg; nickel (Ni) from ND to 17.76 mg/kg; cadmium (Cd) from 1.22 to 5.58 mg/kg; lead (Pb) from 0.17 to 0.98 mg/kg; iron (Fe) from 19.99 to 84.71 mg/kg; calcium (Ca) from 35.15 to 198.53 mg/kg; potassium (K) from 35.31 to 105.19 mg/kg; and magnesium (Mg) from 18.66 to 46.07 mg/kg. The percentage recoveries ranged from 80.5% to 120%, indicating good accuracy and repeatability of the analytical procedure. The study also found significant variations among the six metals, suggesting that geographic origin influences metal levels in rice. Notably, while there was no significant difference between cadmium and calcium concentrations, the levels of nonessential metals, cadmium, lead, and nickel, exceeded the recommended limits set by WHO/FAO. Based on these findings, the study recommends careful handling of rice during transportation, marketing, storage, and cultivation to minimize exposure to toxic metals.

## 1. Introduction

### 1.1. Background of the Study

Over the past few decades, industrial development and globalization have caused significant environmental pollution by discharging heavy metal ions and metalloids such as copper(II) (Cu^2+^), zinc(II) (Zn^2+^), mercury(II) (Hg^2+^), nickel(II) (Ni^2+^), lead(II) (Pb^2+^), cadmium(II) (Cd^2+^), cobalt(II) (Co^2+^), iron(II) (Fe^2+^), chromium(II) (Cr^2+^), and arsenic(III) (As^3+^) into our ecosystems [1–3]. This has led to heavy contamination of soils, water, and crops, which poses a significant threat to human health. These heavy metals are chemically stable and tend to accumulate in crops, ultimately entering the human body through the food chain [4, 5]. Human beings are at a high risk of exposure to these life‐threatening heavy metals, which can cause severe damage to the body. Heavy metal pollutants enter the water and soil environment through various human and agricultural activities including the discarding of electronic and electric waste, chemical fertilizers, mining, pesticides, sewage sludge, industrial processing, and automobile exhaust [6–8].

Different types of toxic heavy metals can cause varying degrees of damage to the human body, leading to central and peripheral neurological damage, cardiovascular disease, birth defects, placental development disorders, and other illnesses [9–13]. Heavy metals are considered trace elements because they are present in trace concentrations (μg/L range to less than 10 mg/L) in various environmental matrices [14]. Among the different types of cereals such as wheat (*Triticum aestivum*), rice (*Oryza sativa*), barley (*Hordeum vulgare*), and maize (*Zea mays*), rice is one of the most widely consumed cereals globally by more than half of the world’s population [15–17]. Rice is a significant source of energy, vitamins, mineral elements, and amino acids (rare) for humans and is the most important food in the world, supplying as much as half of the daily calories of the world population [18, 19]. However, rice is also a major source of trace amounts of essential and nonessential heavy metal exposure [20–22].

Based on ancient and archaeological evidence, *Oryza sativa* is believed to have originated in the foothills of the Himalayas in the North and the hills in the North‐East of India, while *Oryza glaberrima*, the African rice, originated in the delta of River Niger in Africa [23–25]. Since the rice plant is extremely adaptable to the indigenous environment and because humans have succeeded in modifying the local agroecosystem, rice can now be grown in many different locations and a variety of climates [24]. The genus Oryza has more than 25 species of rice. *Oryza sativa*, the predominant rice species, is said to have originated in Southeast Asia. The native plant *Oryza glaberrima*, which was cultivated as the primary staple crop in Western Africa before now, has nearly entirely been replaced by *Oryza sativa* in Africa [26].

Rice is a relatively new crop in Ethiopia, introduced only in the 1970s. Unlike other African and Asian countries where it is a traditional staple food, rice is not a chief food staff in Ethiopian culture [26, 27]. However, it has become a high‐potential crop for emergency and food security purposes in the country. Ethiopian farmers have quickly adapted to growing rice in many regions and over large areas. Varieties of rice are also imported into Ethiopia annually, in addition to locally cultivated ones. Unfortunately, rice is an inadequate source of many essential micronutrients and vitamins, which can lead to deficiencies in developing countries like Ethiopia [11, 28, 29]. For instance, rice lacks vitamin A, so people who consume large amounts of rice are at risk of becoming deficient in this essential vitamin. Additionally, rice may also contain trace amounts of toxic heavy metals, which can pose health risks to the community if ingested [21, 22].

Many communities that consume rice as a staple food are unaware of the levels of essential and nonessential metals in commercially available imported and Ethiopian rice in Jimma. Various countries use different fertilizers during rice production, which may lead to the presence of trace amounts of essential and nonessential metals in the yield. In addition, rice products may become contaminated with toxic heavy metals during importation and while on the market, which can pose health risks to humans upon consumption. The increasing demand for safe food has prompted research into the risks associated with consuming food contaminated with pesticides, heavy metals, and toxins. Heavy metals are potential environmental contaminants that can cause human health problems if present in excessive amounts in the food we eat. Despite the community’s increasing desire to consume rice and the associated risks, no research has been conducted to analyze the levels of essential and nonessential metals in rice samples. Thus, the objective of this study is to examine the quantity of essential and nonessential heavy metals present in chosen local and imported rice in Jimma‐Ethiopia. The study analyzes the levels of Cr, Ni, Cd, Pb, Fe, Ca, K, and Mg and compares them with national and global standards to provide suitable recommendations for the community.

### 1.2. Novelty of the Study

This study advances the field of analytical chemistry by demonstrating its effective application in a new regional context, providing a comprehensive analysis that can serve as a reference for future research on rice and similar food commodities in Ethiopia and comparable regions. Unlike previous studies that have primarily focused on rice from other areas or analyzed only a limited set of elements, our research offers a detailed, context‐specific dataset that reflects the unique elemental composition of rice within Ethiopia’s diverse agricultural and processing environments. Importantly, this is the first systematic comparison of local versus imported rice and raw versus cooked samples within this geographic setting. These insights reveal potential elemental disparities and food safety issues specific to Ethiopia. By adopting this targeted approach, our work not only advances analytical methodologies in the regional context but also provides valuable baseline data to support future food security and nutritional assessments. Overall, the focus on Ethiopia and the depth of our analysis distinguish this research from prior studies, representing a meaningful contribution to the regional application of food elemental analysis.

## 2. Materials and Methods

### 2.1. Sample Site

This study was conducted in Jimma town, which is found in the Oromia regional state in the southwest part of Ethiopia. Jimma has a latitude and longitude of (7°40′N, 36°50′E). The geographical representation of the study area is given in Figure S1.

### 2.2. Sample Collection

Rice samples were collected from the Jimma town market. The samples included four different types: Basmati, Jasmine, Dana (all imported), and Chawaka (local). A total of 2 kg of each rice sample was collected from several supermarkets in Jimma Town, making a total of 24 kg (at least three replicates of each sample were collected). The samples were then carefully placed in clean polyethene bags, labeled, and taken to the laboratory for further pre‐treatment. Once arrived at the laboratory, the samples were prepared for analysis.

### 2.3. Chemicals and Reagents

Analytical‐grade chemicals were completely used for digesting the rice samples and standard preparation. HNO_3_ (69% from Sigma‐Aldrich, Germany), HClO_4_ (70% from Merc, Germany), and H_2_O_2_ (30% w/w from Sigma‐Aldrich, Germany) were used as received for rice sample digestion. Stock standard solutions (1000 mg/L) of the metals Ca, Mg, K, Fe, Pd, and Cr were prepared from their nitrate salts (Loba Chemie, Mumbai, India), while Cd standard solution was prepared from Cd (SO_4_)_2_ from the same supplier.

### 2.4. Sample Preparation

To ensure that there were no dust particles on the grains, all raw rice samples (Basmati, Jasmine, Dana [all imported], and Chawaka [local]) were washed with tap water and deionized water. The samples were then dried in an oven at 80°C until they reached a constant weight. Next, the dried rice was ground in a laboratory blender and sieved to remove any large particles. Finally, the rice samples were cooked using the absorption and excess water method.

Samples weighing a combined 24 kg were collected from three stores in Jimma town, representing four different species of rice: Ethiopian‐produced rice (Chawaka), Jasmine, Dana, and Basmati. A total of 2 kg of each sample was collected from each business to make a 3 kg bulk sample. The rice was cooked in 5 L of tap water, and unique samples of four composites were created. Four groups containing 1.5 kg of each type of rice‐cooked and uncooked were created.

The study aimed to investigate the impact of cooking on metal levels in rice samples. Excess water methods was used for this purpose; 125 g of rice sample was cooked using the best excess water method. After the cooking process was completed, the cooked rice samples were ground and allowed to dry until they reached a constant weight. The dried samples were then ground to obtain the required particle size.

### 2.5. Sample Digestion and Standard Preparation

For preparing calibration standards and spiking experiments, stock standard solutions containing 1000 mg/L of metal Ca, Mg, K, Fe, Ni, Cr, Cd, and Pb in 2% HNO_3_ were used. Throughout the experiment for sample preparation, dilution, and rinsing apparatus before analysis, deionized water was used. All glassware used in volume measurement, reagent solution preparation, sample digestion, and sample holding were cleaned before and after use with a metal‐free liquid detergent, rinsed with distilled water, soaked in 20% HNO_3_, rerinsed with deionized water, and dried to avoid contamination [9, 22, 30].

About 0.5 g of powdered and homogenized rice from each sample was obtained and put into a 100‐mL round‐bottom flask. H_2_O_2_ (36%), HClO_4_ (70%), and concentrated HNO_3_ (69%–72%) were added to this. The combination was then heated to the proper temperature on the Kjeldahl digesting device, which was attached to a reflux condenser via the flask. For every rice sample, triplicate digestions were performed in the optimization digestion process until a clear solution was achieved. In triplicate, blank solutions were likewise processed appropriately. Until analysis, the digested and diluted sample solutions were stored in a refrigerator. The metal content of the raw and cooked rice samples was determined using these digestive procedures.

### 2.6. Optimized Digestion Procedure Result

Different digestion procedures were tested using HNO_3_, HClO_4_, and H_2_O_2_ with varying volumes, reagents, temperatures, and times to optimize the process (Table S1).

### 2.7. Instrument Working Condition

The AAS instrument was equipped with a deuterium arc background corrector and a standard air acetylene flame system. After optimizing the parameters such as burner and lamp alignment, slit width, and wavelength, the external calibration curve was used to obtain the maximum signal intensity. For each metal, hollow cathode lamps were used at their respective primary source line under the manufacturer’s recommended conditions. The operating conditions of the FAAS instrument for each analyte are provided in Table S2.

### 2.8. Instrumental Calibration

**FIGURE 1:**
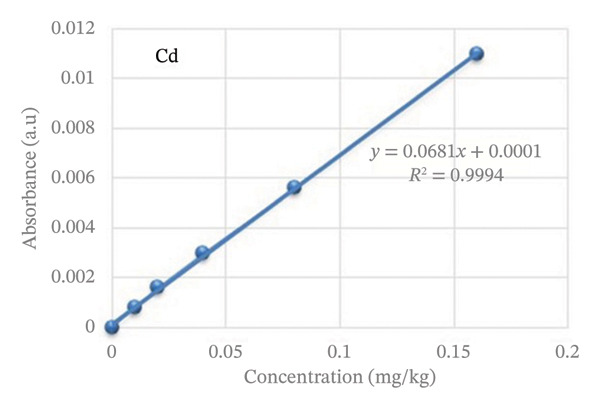
The calibration curve of Cd determination.

The quality of the data produced by AAS (VGP‐210 FAAS) for vital and nonessential metal analysis was significantly impacted by standard solution preparation techniques and calibration. Intermediate standard solutions of 10 mg/L were prepared from stock standard solutions that contain 1000 mg/L. The standard was diluted with deionized water to obtain five working standards for each metal. Calibration curves were drawn to determine the concentration of essential and nonessential metals in the solution for the rice sample. The representative calibration curve for the standards is given in Figure 1, while the remaining were added to the supporting information (Figures S2–S8). The concentration of working standards and the value of the correlation coefficient of the calibration graph for each metal are listed in Table S3.

To find the metal concentration in the sample solution, calibration curves plotting the absorbance against concentration were created. The Cd calibration curve was provided as an illustration, while the other is included in the Supporting Information (available here).

With coefficients of determination (*R* ^2^) ranging from 0.9992 to 0.9998, all more than the acceptable threshold of 0.995 for the linearity of the regression line, calibration curves for a variety of concentrations demonstrated strong linearity (Figures S1‐S7).

### 2.9. Method of Validation

#### 2.9.1. Precision and Accuracy

The recoveries of the cooked and uncooked rice samples ranged from 80.15% to 119.9%. They were all within the acceptable range of 80%–120%. There was a % RSD of 0.22–18.15 for both cooked and uncooked rice samples.

The %RSD values of Cr in Jasmin uncooked sample, Cd in Jasmin cooked, Cd in Jasmin raw, Ca in Jasmin uncooked, and Mg in Chawaka cocked sample were 0.22, 0.86, 1.48,1.96, and 1.95, respectively. This indicates that the measurement of those metals in this sample was more precise than the other. According to the required control limit, the methods had good precision and accuracy (Table 1).

**TABLE 1:** Recovery (%) obtained for authentication of the optimized procedure after spiking with standard solutions (*n* = 3).

Metal	Type of sample	Concentration of USS. mean ± SD deviation (mg/L)	Concentration of SS. mean ± SD deviation (mg/L)	Amount added (mg/L)	Recovery (%)	%RSD
Cr	CHC	< LoQ	< LoQ	—	—	—
	CHR	0.01 ± 0.0004	0.198 ± 0.02	0.2	94.00	4.00
	BC	0.0501 ± 0.0013	0.290 ± 0.010	0.2	119.90	2.58
	BR	0.0521 ± 0.0035	0.25 ± 0.012	0.2	98.95	6.7
	JC	0.1055 ± 0.0069	0.31 ± 0.021	0.2	102.25	6.54
	JR	0.0816 ± 0.0002	0.28 ± 0.003	0.2	99.20	0.22
	DC	0.1536 ± 0.0085	0.34 ± 0.0013	0.2	108.20	0.55
	DR	0.1298 ± 0.0110	0.33 ± 0.0012	0.2	100.01	8.40

Ni	CHC	< LoQ	< LoQ	—	—	—
	CHR	0.0519 ± 0.0034	0.23 ± 0.0210	0.2	89.50	6.55
	BC	0.0817 ± 0.0041	0.31 ± 0.1000	0.2	114.50	5.01
	BR	0.0302 ± 0.0025	0.21 ± 0.0010	0.2	89.90	8.27
	JC	0.1069 ± 0.0016	0.32 ± 0.0010	0.2	106.50	1.49
	JR	0.1228 ± 0.0149	0.32 ± 0.0020	0.2	98.60	12.13
	DC	0.1776 ± 0.0242	0.34 ± 0.0010	0.2	81.20	13.62
	DR	0.1743 ± 0.0324	0.36 ± 0.0020	0.2	92.85	12.25

Cd	CHC	0.0122 ± 0.0013	0.19 ± 0.0010	0.2	88.90	10.65
	CHR	0.028 ± 0.0013	0.22 ± 0.0030	0.2	96.00	4.62
	BC	0.0427 ± 0.0049	0.241 ± 0.0020	0.2	99.15	11.47
	BR	0.0358 ± 0.0025	0.22 ± 0.0030	0.2	92.00	6.98
	JC	0.0519 ± 0.0043	0.232 ± 0.0020	0.2	90.05	0.86
	JR	0.0558 ± 0.0008	0.251 ± 0.0002	0.2	97.60	1.48
	DC	0.0369 + 0.0012	0.21 ± 0.0005	0.2	86.55	3.20
	DR	0.0531 + 0.0018	0.25 ± 0.0003	0.2	98.50	3.33

Pb	CHC	0.0066 ± 0.0004	0.19 ± 0.0002	0.2	91.70	6.06
	CHR	0.00978 ± 0.0003	0.198 ± 0.0012	0.2	94.11	3.06
	BC	0.0052 ± 0.0003	0.215 ± 0.0002	0.2	104.90	4.60
	BR	0.0041 ± 0.0001	0.198 ± 0.0018	0.2	96.95	2.43
	JC	0.0017 ± 0.0001	0.162 ± 0.0020	0.2	80.15	7.06
	JR	0.0084 ± 0.0010	0.185 ± 0.0300	0.2	88.30	11.90
	DC	0.0057 ± 0.0001	0.21 ± 0.0020	0.2	102.50	1.70
	DR	0.0088 ± 0.0005	0.19 ± 0.0003	0.2	90.60	6.37

Fe	CHC	0.4443 ± 0.0413	0.96421 ± 0.1000	0.5	103.98	9.28
	CHR	0.7701 ± 0.0034	1.21 ± 0.2000	0.5	87.98	0.44
	BC	0.3062 ± 0.0383	0.801 ± 0.1100	0.5	95.89	12.50
	BR	0.7701 ± 0.0413	1.04 ± 0.2100	0.5	97.39	5.36
	JC	0.0646 ± 0.0030	0.53 ± 0.3000	0.5	89.35	4.659
	JR	0.623 ± 0.1037	1.23 ± 0.4000	0.5	92.14	16.64
	DC	0.6683 ± 0.0125	1.13 ± 0.1000	0.5	92.34	1.87
	DR	0.3515 + 0.0200	0.81 ± 0.1300	0.5	91.70	5.68

Ca	CHC	44.42 ± 4.1295	47.88 ± 2.1300	0.4	86.50	9.29
	CHR	77.0118 ± 3.3944	80.98 ± 2.3200	0.4	99.20	4.40
	BC	30.6212 ± 1.8417	33.91 ± 2.7500	0.4	82.22	6.01
	BR	19.9853 ± 0.6789	23.5 ± 1.5000	0.4	87.86	3.39
	JC	8.13 ± 0.3542	11.31 ± 0.5100	0.4	80.50	4.35
	JR	59.3443 ± 1.1646	63.21 ± 3.1200	0.4	96.64	1.96
	DC	0.8285 ± 0.0020	1.2 ± 0.0500	0.4	92.88	2.40
	DR	0.8471 ± 0.0015	1.3 ± 0.0200	0.4	113.15	1.77

K	CHC	0.4074 ± 0.0013	0.81 ± 0.0010	0.4	100.06	3.19
	CHR	0.3531 ± 0.0050	0.68 ± 0.0013	0.4	81.72	1.40
	BC	1.0519 ± 0.0300	1.39 ± 0.0020	0.4	84.52	2.85
	BR	1.03 ± 0.0800	1.39 ± 0.0010	0.4	90.00	7.76
	JC	0.358 ± 0.0050	0.71 ± 0.0003	0.4	88.00	1.39
	JR	0.6667 ± 0.0100	0.99 ± 0.0011	0.4	80.82	1.50
	DC	0.679 ± 0.0120	1.02 ± 0.00189	0.4	85.25	1.76
	DR	0.5802 ± 0.0500	0.96 ± 0.0006	0.4	94.95	8.60

Mg	CHC	0.3333 ± 0.0065	0.724 ± 0.0003	0.4	97.67	1.95
	CHR	0.1866 ± 0.0093	0.541 ± 0.0010	0.4	88.60	4.98
	BC	0.4542 ± 0.0163	0.831 ± 0.0012	0.4	94.20	3.588
	BR	0.1604 ± 0.0049	0.541 ± 0.0210	0.4	95.15	3.05
	JC	0.526 ± 0.0025	0.891 + 0.0220	0.4	98.20	5.89
	JR	0.1963 ± 0.0099	0.59 ± 0.0030	0.4	98.42	5.04
	DC	0.397 + 0.0200	0.75 ± 0.0100	0.4	88.25	5.03
	DR	0.3344 + 0.0120	0.79 ± 0.0210	0.4	113.90	3.58

*Note:* CHC = Chawaka cooked, CHR = Chawaka raw, USS = unspiked sample.

Abbreviations: BC = Basmati cooked, BR = Basmati raw, JC = Jasmin cooked, JR = Jasmin raw, SS = spiked sample.

### 2.10. Statistical Analysis

All analyses and extractions were performed in triplicate, and the data are expressed as mean ± standard deviation (SD).

#### 2.10.1. Detection Limit

IDL, LoD, and LoQ for each metal analyte were calculated from the instrument response of three replicate blank sample analyses (Table S4). Blank samples, which were similar to the composition of the matrix with samples without analyte, were digested.

## 3. Results and Discussion

### 3.1. Concentration of Essential and Nonessential Metals in Rice

The amount of rice consumed, its geographic location, the metal sources in the area where it is cultivated, the type of rice consumed, and the conditions of the soil and irrigation water used for production all affect how exposed rice is to metals.

The concentration of some essential and nonessential metals in cooked and uncooked rice that was cultivated in Ethiopia and imported to Ethiopia was measured. Not all the concentrations of the metal analyzed were detected except Cr, and Ni was detected in Chawaka cooked sample. The result is summarized in Table 2.

**TABLE 2:** Concentration of essential and nonessential metals in rice (*n* = 3, mg/kg).

Samples	Cr	Ni	Cd	Pb	Fe	Ca	K	Mg
CHC	0.1 ± 0.04	1.59 ± 0.34	1.22 ± 0.42	0.66 ± 0.04	44.43 ± 4.13	42.53 ± 2.95	40.74 ± 3.70	33.33 ± 0.65
CHR	< LoQ	< LoQ	2.81 ± 0.13	0.98 ± 0.03	77.01 ± 3.39	77.18 ± 9.44	35.31 ± 1.67	18.66 ± 0.93
BC	5.21 ± 0.35	3.02 ± 0.25	3.58 ± 0.65	0.41 ± 0.01	19.99 ± 0.68	198.53 ± 7.89	105.19 ± 5.01	46.07 ± 0.49
BR	5.01 ± 1.29	8.17 ± 0.41	4.27 ± 0.49	0.52 ± 0.024	30.62 ± 11.40	62.12 ± 4.17	103.00 ± 0.64	45.42 ± 1.63
JC	10.55 ± 0.69	10.69 ± 0.16	5.19 ± 0.43	0.17 ± 0.06	61.46 ± 3.01	81.00 ± 5.42	35.80 ± 7.71	20.02 + 0.02
JR	8.16 ± 0.18	12.28 ± 1.49	5.58 ± 0.83	0.84 ± 0.10	62.30 ± 10.70	93.43 ± 6.46	66.67 ± 3.70	19.63 ± 0.99
DC	12.98 ± 1.68	17.43 ± 3.24	5.31 ± 1.77	0.88 ± 0.51	66.83 ± 11.25	82.85 ± 5.48	67.90 ± 9.32	39.70 ± 1.31
DR	15.36 ± 0.85	17.76 ± 2.42	3.69 ± 0.12	0.57 ± 0.10	35.15 ± 10.37	84.71 ± 7.01	58.02 ± 4.27	33.44 ± 4.94
WHO/FAO	20	0.20	0.30	0.30	500	200	300	500
Ref.	[30]	[31]	[10, 11, 13]	[13, 30]	[11, 30]	[11, 13]	[10, 13]	[10, 11, 30]

Concentrations in mg kg^−1^ of metal in the digested samples were calculated using the following expression:

(1) M=C×VW,

where [*M*] = total metal concentration (in mg kg^−1^), *C* = concentration of the metal in the digested samples (in mgL^−1^), *V* = final volume of the digested sample solution (50 mL) changed to *L* (0.05 L), and *W* = weight/mass of the digested sample (0.5 g) changed to kg (0.0005 kg).

#### 3.1.1. Level of the Concentration of Metal in Cooked and Raw Rice Samples

From the whole rice analyzed for metal level determinations, Fe, Ca, and K were the highest in concentration, while Pb and Cr were the lowest (Figure 2). The concentration of Ni and Cd was not detected in the Chawaka raw sample because it was below the instrument detection limit. Concentrations of metals are listed in order of decreasing for Chawaka cooked: Cr < Pb < Cd < Ni <Mg < K < Ca < Fe. The lowest concentration in Chawaka cooked was Cr (0.1 mg/kg), and the highest concentration was Fe (44.43 mg/kg). On the other hand, concentrations of metals were also arranged in Chawaka raw: Pb < Cd < Mg < K < Ca < Fe. Ni and Cr were not detected in Chawaka raw due to the below detection limit. The lowest concentration of Chawaka raw was Pb (0.98 mg/kg), whereas the highest concentration was Fe (77.01 mg/kg). The trend in Chawaka raw and Chawaka cooked is not the same as Ni and Cd that were not detected in Chawaka raw were detected in Chawaka cooked and the order of Ca and Fe were exchanged. There is some increment of concentration of Ca in Chawaka cooked. Therefore, the difference between Chawaka raw and Chawaka cooked is not significant (Figure 2(a)).

FIGURE 2Comparison of the concentration of essential and nonessential metals in cooked and raw rice samples (a) Chawaka, (b) Basmati, (c) Jasmine, and (d) Dana.

**Figure:**
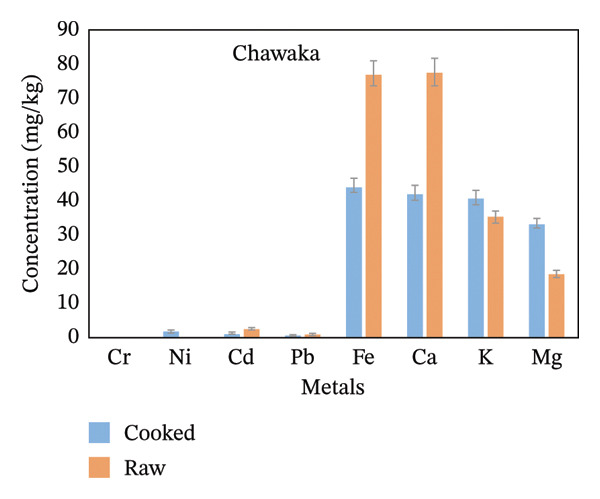
(a)

**Figure:**
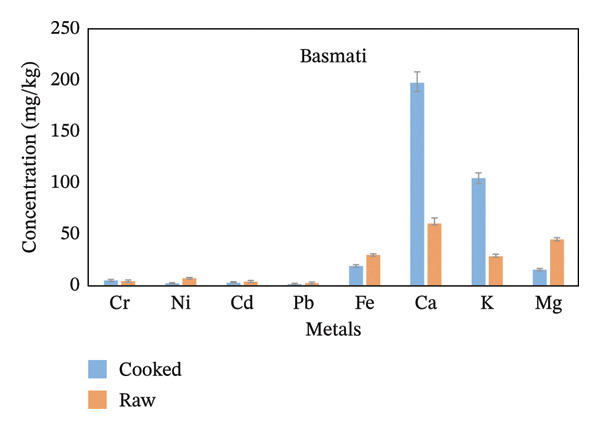
(b)

**Figure:**
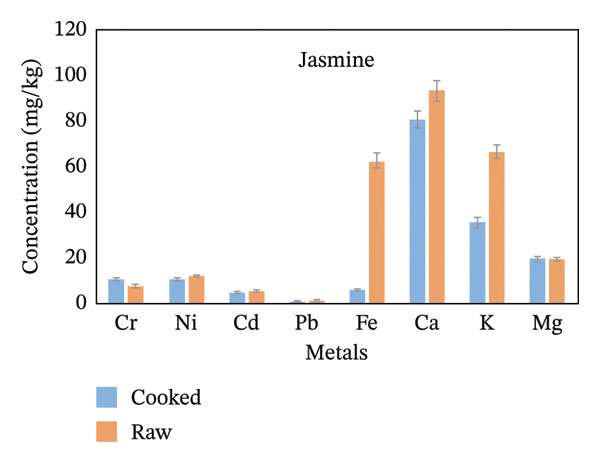
(c)

**Figure:**
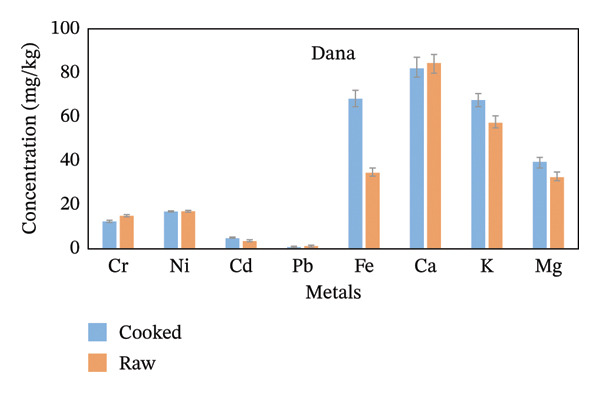
(d)

Basmati cooked: Pb < Ni < Cd < Cr < Mg < Fe < Ca. The lowest concentration of Basmati cooked was Pb (0.41 mg/kg), whereas the highest concentration was Ca (198 mg/kg). Basmati raw: Pb < Cd < Cr < Ni < K < Fe < Mg < Ca (Figure 2(b)). The lowest concentration of Basmati raw was Pb (0.52 mg/kg), whereas the highest concentration was Ca (62.12 mg/kg). The order of the concentration of Ni, Cd, Cr, Fe, K and Mg is not the same in Basmati cooked and Basmati raw. Therefore, their difference is not significant.

Jasmine cooked Pb < Cd < Cr < Ni < Mg < Fe < K < Ca. The lowest concentration of jasmine cooked was Pb (0.17 mg/kg), whereas the highest concentration was Ca (81.00 mg/kg). Jasmine raw: Pb < Cd < Cr < Ni < Mg < Fe < K < Ca (Figure 2(c)). The lowest concentration of Jasmine raw was Pb (0.84 mg/kg), whereas the highest concentration was Ca (93.43 mg/kg). The order of the concentration of essential and nonessential metals in a Jasmine cooked and Jasmine raw is the same, so the difference is significant.

Dana cooked Pb < Cd < Cr < Ni < Mg < Fe < K < Ca. The lowest concentration in a Dana cooked was Pb (mg/kg), whereas the highest concentration was Ca (82.5 mg/kg). Dana raw: Pb < Cd < Cr < Ni < Mg < Fe < K < Ca. The lowest concentration in a Dana raw was Pb (0.57 mg/kg), whereas the highest concentration was Ca (84.71 mg/kg). The order of the concentration of essential and nonessential metals in Dana cooked and Dana raw is the same, so the difference is significant (Figure 2(d)).

### 3.2. Analysis of the Concentration of Essential and Nonessential Metals in Cooked and Raw Rice

The elements Ca, Mg, K, Cr, Fe, Ni, Cd, and Pb were analyzed in the rice samples. Among these, Ca, Mg, Mn, Fe, Cu, and Zn were determined quantitatively in all of the rice samples (Table 2). Even though they were detected, the elements Cr, Ni, and Cd were all below the LOQ of the method, and hence were not quantified accurately. In contrast, Pb was not detected in any of the rice samples.

#### 3.2.1. Concentration of Cr in Cooked and Raw Rice Samples

Numerous animal species are susceptible to chromium deficiency, which can lead to reduced glucose tolerance in the presence of normal levels of circulating insulin and, in extreme situations, a state resembling diabetes. Compared to chromium (VI), chromium (III) is far less hazardous. In this study, the mean concentration of chromium was in the range of 0.1–15.6 mg/kg. The results were significantly different at (*p* < 0.05) The previously reported value of the concentration of chromium in imported rice was 2.2–3.12 mg/kg, and in Ethiopian rice, it was in the range of 2.32–4.82 mg/kg [12, 27]. Fitige Birihen (2020) reported a high concentration of chromium in rice from Central and South Gondar zones in the range of 19.9–30.5 g/kg. The mean concentration of Cr (Nd‐0.1 mg/kg) in Ethiopian rice in the present study was lower than in the previous studies [27], whereas the mean concentration of chromium (5.21–15.36 mg/kg) in imported rice of the present study was higher than that previously reported by researchers [27]. This variation may be due to concentrations in soil, fertilizer, water supply, and transportation. The concentration of Cr in Chawaka raw was not detected, while it was 0.1 mg/kg in Chawaka coked. This indicates that the tap water which was used for cooked rice contained Cr. The concentration of Cr in Basmati and Jasmine was higher in cooked than in the raw samples. It may be that there was a transfer of concentration Cr from the tap water to the sample. In the case of the Dana sample, the Cr was higher in raw rice than cooked rice. Generally, the concentration of chromium in rice samples was below the permissible limit of WHO/FAO, so it has no adverse health effect on consumers.

#### 3.2.2. Concentration of Ni in a Cooked and Raw Rice Sample

At very trace levels, Ni is considered an essential trace element. It acts as an activator of some enzyme systems, but its toxicity at higher levels is more prominent. High levels of Ni can cause respiratory problems, and it is carcinogenic. Nickel is known to be responsible for cancer (oral and intestinal), depression, heart attacks, kidney dysfunction, low blood pressure, malaise, muscle tremors and paralysis, nausea, skin problems, and vomiting [29, 31]. The concentration of Ni in this study ranged from 1.59 to 17.76 mg/kg. It was lower in the sample of Chawaka cooked rice (1.59 mg/kg) sample and higher in Dana raw rice (17.76 mg/kg). Except in a sample of Chawaka raw was not detected, whereas in another sample, the concentration of Ni was higher in the raw rice sample than in the cooked sample. The result was significantly different (*p* = 0.00). The concentration of Ni in imported rice (Jasmin and Bismuth rice) reported was in the range of 2.5–75.1 mg/kg and Ethiopian rice (41.5–69.7 mg/kg), which was higher than that reported in the current study. WHO/FAO reported that the permissible limit of nickel in rice was 0.2 mg/kg. In a current study except in a sample of Chawaka cooked, all are above the permissible limit. Therefore, it has adverse health effects on human beings. The source of Ni in this sample may be due to the sewage and industrial wastewater used for irrigation in agriculture activities, transportation, storage, marketing, and processing stages.

#### 3.2.3. Concentration of Cd in a Cooked and Raw Rice Sample

Accumulation of Cd in the kidney leads to high blood pressure and renal diseases. Its accumulation also leads to damaging the nerve cells, inhibiting the release of acetylcholine and activation of choline esterase enzyme, resulting in a tendency for hyperactivity of the nervous system.

Guade et al. reported that the source of the Cd in rice is most probably from the soil as various regions of Bangladesh have been shown to contain elevated Cd levels in soil [32]. In addition, Cd can be added to the soil via fertilizers used during cultivation and thereafter absorbed via the roots of the rice plants. In this study, the level of the concentration of Cd was from 1.22 to 0.58 mg/kg. It was low in a sample of Chawaka cooked (1.22 mg/kg) and high in Jasmine raw (5.58 mg/kg). The concentration of Cd in the Dana raw rice sample was higher than the concentration of Cd in the Dana cooked sample. The results were significantly not different (*p* > 0.05). In other samples, the concentration of the Cd was higher in raw rice than in a cooked rice sample. It may be due to the loss of some concentration when cooked in the form of evaporation. Many epidemiological studies conducted in Matlab have assumed that the high concentrations of Cd in the blood and urine of pregnant women and children originate mainly from the rice‐based diet. WHO/FAO reported that the permissible limit of concentration of Cadmium in rice was 0.3 mg/kg [32]. The mean concentrations of Cd in current studies were higher than the permissible limit. This indicated that it was polluted by cadmium; it has an adverse health effect on human beings, who consume this rice. Therefore, care should be taken when consumers use a rice‐based diet.

#### 3.2.4. Concentration of Pb in a Cooked and Raw Rice Sample

In addition to causing decreased fertility, lead also causes cancer and other minor effects such as vomiting, nausea, and headaches. High Pb levels can cause severe brain and kidney damage, miscarriages in pregnant women, damage to sperm‐producing organs in men, and eventually death [33, 34]. In paint industries, lead is an essential component, which is released into the air, water, and soil during gasoline combustion. As a result of the atmospheric deposition of Pb on soil, food, such as rice, can contain high levels of the metal. The range of the concentration of Pb in the current study was from 0.17 mg/kg in the Jasmine cooked sample to 0.88 mg/kg in the Dana cooked sample. The concentration of Pb was higher in Dana cooked than Dana raw sample, whereas the other concentration was higher in raw than in cooked samples of rice. The mean concentration of lead reported in Ethiopian rice by Ayanaw et al. (0.8–5.3 mg/kg) and Fitige Birihen, 2020 (1.24–1.49 mg/kg) were higher than the recommended limit WHO/FAO (0.3 mg/kg) [27, 29]. The concentration of lead (0.17 mg/kg) in this study in a Jasmine cooked sample was lower than the permissible limit by WHO/FAO (0.3 mg/kg), so it has no adverse health effect at this level. However, in another sample of rice (Basmati, Dana, and Chawaka), it was above the recommended limit of FAO/WHO, so it has an adverse health effect on consumers. The concentration of Pb in this study was lower than the other three nonessential Cr, Ni, and Cd metals.

#### 3.2.5. Concentration of Fe in a Cooked and Raw Rice Sample

Iron plays a vital function in rice photosynthesis. It may impede K absorption because of its deficiency. The youngest rice leaves show the very first symptoms of its deficiency because of their immobile nature. The occurrence of iron poisoning in plants is associated with high uptake of Fe^+2^ by roots and its transport to leaves and through transpiration. Excess Fe^+2^ creates free radicals that change cell structure and damage cell membranes, DNA, and proteins. The levels of the concentration of Iron in the present study ranged from 19.99 to 84.71 mg/kg. The concentrations of Fe (19.99 mg/kg) were low in the Basmati cooked sample and highest (84.71 mg/kg) in the Dana raw sample. The concentrations of Fe in this study were higher in a raw sample of rice than in a cooked sample of rice. The result was significantly different (*p* = 0.00). The concentration decrease in a cooked sample may be because the tap water may have a very low concentration of Fe or it may be loosed when cooked. The permissible limit of concentration of Fe by WHO/FAO was 500 mg/kg. All the concentration of Fe in this study was below the permissible limit. So, it has no health effect on a human being.

#### 3.2.6. Concentration of Ca in a Cooked and Raw Rice Sample

Adverse symptoms of calcium excess can include renal failure, soft tissue calcification, irritability, headache, and various other clinically evident signs.

The concentration of Ca in this study was higher than the other metals. It ranged from 42.53 to 198.653 mg/kg. The lowest concentration (42.53 mg/kg) was in a sample of Chawaka cooked rice, whereas the highest concentration (198.653 mg/kg) was in a Basmati cooked rice sample. The concentration of Ca (42.53 mg/kg) in Chawaka cooked < Chawaka raw (77.18 mg/kg), Basmati cooked (198.15 mg/kg) > Basmati raw(62.12 mg/kg), Jasmine cooked (61.46 mg/kg) < Jasmin raw(62.30 mg/kg), Dana cooked (66.83 mg/kg) > Dana raw (35.15 mg/kg). The concentration of Ca was higher in Basmati and Dana cooked rice samples than in raw sample, which may be due to the polluted tap water used for cooking, whereas it was higher in a raw Jasmine and Chawaka rice sample than in a cooked rice sample. The results were not significantly different (*p* > 0.005). The permissible limit of the concentration of Ca in rice by WHO/FAO was 200 mg/kg. All the concentration of the Ca was below the permissible limit.

#### 3.2.7. Concentration of K in a Cooked and Raw Rice Sample

Potassium is an essential factor in optimizing root development, enhancing plant vigor, reducing lodging, encouraging cell division, supplying osmotic pull, helping to neutralize organic acid, and boosting seed resistance to pests and diseases, helping in maintaining metabolism. The ranges of the concentration of potassium in the current study were from 35.31 to 105.19 mg/kg. The lowest concentration of potassium (35.31 mg/kg) was in the Chawaka raw rice sample, whereas the highest concentration (105.19 mg/kg) was in a Basmati raw rice sample. The concentration of K in a rice sample was: Chawaka cooked (40.74 mg/kg) > Chawaka raw (35.31 mg/kg), Basmati cooked (105.19 mg/kg > Basmati raw (103.0 mg/kg), Jasmine cooked (35.80 mg/kg) < Jasmine raw.

Raw (66.67 mg/kg), Dana cooked (67.90 mg/kg) > Dana raw (58.02 mg/kg). In the sample of this study, the concentration of K is higher in a cooked sample of rice than in the raw sample of rice; only the concentration of K in Jasmine raw was greater than the Jasmine cooked. The result was significantly not different (*p* = 0.00036). In a current study, the concentration of K was below the permissible limit WHO/FAO (300 mg/kg).

#### 3.2.8. Concentration of Mg in a Cooked and Raw Rice Samples

The level of magnesium in this study ranged from 18.66 to 46.07 mg/kg. The lowest concentration of magnesium (18.66 mg/kg) was in a Chawaka raw sample, whereas the highest concentration was in a Basmati (46.07 mg/kg) cooked sample. In this study, the concentrations of Mg were Chawaka cooked (33.33 mg/kg) > Chawaka raw (18.66), Basmati cooked (46.07 mg/kg) > Bismuth raw (45.42 mg/kg), Jasmine cooked (20.02 mg/kg) > Jasmine raw (19.63 mg/kg), Dana cooked (39.70 mg/kg) > Dana raw (33.44 mg/kg). The concentration of Mg in this study was higher in the cooked rice sample than in the raw rice sample. The concentration of Mg in this study was lower than the permissible limit by WHO/FAO of 500 mg/kg.

### 3.3. Comparisons of the Concentration of Essential and Nonessential Metals Imported Rice With Ethiopian Rice

Plant uptake of heavy metals from soil and heavy metal contamination of foods during the harvesting, transportation, storage, marketing, and processing stages are the main sources of heavy metals in foods or the frequent use of water contaminated with heavy metals in agricultural fields, leading to soil pollution and gradually enriching the soil with heavy metals.

From Table 3, the concentrations of the metals in Ethiopian rice were compared with imported rice. The concentration of Cr (ND‐0.1 mg/kg) in Ethiopian rice was smaller than in imported rice (5.01–15.6 mg/kg); the level of Ni (ND‐1.59 mg/kg) in Ethiopian rice was smaller than in imported rice (3.02–17.76 mg/kg). The level of Cd in Ethiopian rice was between 1.22 and 2.81 mg/kg, whereas in imported rice, it was between 3.58 and 5.58 mg/kg. Therefore, Cd in Ethiopian rice was lower than in imported rice. The concentration of Ni, Cr, and Cd in imported rice is higher than in Ethiopian rice. This may be due to storage, transportation, and marketing. The concentration of Pb (0.66 kg/kg) in cooked Ethiopian rice is higher than in Bismuth cooked (0.41 mg/kg), Basmati raw (0.52 mg/kg), Jasmin cooked (0.17 mg/kg), and Dana raw rice (0.57 mg/kg). The concentration of Fe in Ethiopian rice, Chawaka cooked (44.43 mg/kg) was higher than in the imported rice of Bismuth cooked (19.99 mg/kg) and Bismuth raw (30.62 mg/kg), and it was lower than in Jasmine and Dana rice. The concentration of Ca, K, and Mg in Ethiopian rice was generally higher than in imported rice.

**TABLE 3:** Comparison of the concentration metal (mg/kg) in Ethiopian rice with imported rice.

Metals	Rice samples							
	Rice cultivated in Ethiopia		Imported rice					
	CHC	CHR	BC	BR	JC	JR	DC	DR
Cr	0.10	ND	5.21	5.01	10.55	8.16	12.98	15.36
Ni	1.59	ND	3.02	8.17	10.69	12.28	17.43	17.76
Cd	1.22	2.81	3.58	4.27	5.19	5.58	5.31	3.69
Pb	0.66	0.98	0.41	0.52	0.17	0.84	0.88	0.57
Fe	44.43	77.01	19.99	30.62	61.46	62.30	82.88	84.71
Ca	42.53	77.18	198.53	62.12	61.46	62.30	66.83	35.15
K	40.74	35.31	105.19	103.0	35.80	66.67	67.90	58.02
Mg	33.31	18.66	46.07	45.42	20.02	19.63	39.70	33.44

### 3.4. Comparisons of the Current Study With Different Literature Review

It is important to compare the amounts of heavy metals obtained from the analyses of rice samples from the commercially available rice in Jimma town with the amounts cited in the literature from the countryside and other countries. This comparison helps to indicate the differences in composition and the existence of deviation from certain guidelines (Table 4).

**TABLE 4:** Comparisons of the concentration of essential and nonessential metal (mg/kg) in the current study with different literature reviews.

Location	Cr	Ni	Cd	Pb	Fe	Ca	K	Mg	References
Ethiopia	ND‐15.36	ND‐17.76	1.22–5.58	0.17–0.98	19.99–84.71	35.15–198.53	35.31–105.19	18.66–46.07	Current study
Malawi	BDL	—	BDL	BDL	1.83–2.49	5.19–7.81	216.02–268.78	30.22–40.32	[15]
Thailand	2.6–237	0.3–206	ND‐0.43	0.5–30.3	1.3–197	0.5–14.3	0.20–13.60	ND‐15.3	[17]
Nigeria	14.17	0.90	0.45	0.60	14.71	—	—	—	[19]
Ethiopia	2.32–4.82	41.5–69.7	0.45–2.54	0.8–3.8	41.3–113	205–427	1100–3020	99.5–2250	[20]
Iran	—	—	—	—	0.716	8.43	8.16	11.03	[21]
Ethiopia	< LOD	< LOD	< LOD	< LOD	168	45.2	—	561	[35]

This comparison helps to identify the differences in composition and if there exists a deviation from certain guidelines. As the comparison in Table 4 shows, most of the values reported in the literature are compared with the present study. K (216.02–268.78 mg/kg) in the literature is higher to some extent than in the present study (35.31–105.19 mg/kg) and to a similar extent in the literature (1100–3020 mg/kg) to the present study. The level of the concentration of potassium (1100–3020 mg/kg) in the present study was greater than the one reported in the literature (0.2–13.6 mg/kg). The concentrations of Mg in the present study were similar to some extent to the reported literature, and it was a lower greater extent than that reported in the literature. The concentration of essential metals (Ca, K, and Mg) is not reported. The higher extent of concentration of Ca, K, and Mg in Ethiopian rice of the literature than in the present study was reported, which was due to this metal being mobile into plant tissue. The concentration of Fe in the present study was a higher greater extent and lower than the greater extent than in the literature. The concentration of Pb in the current study was lower to some extent than in the literature to a similar extent. The concentrations of Cd in the present study were lower to some extent than most of the literature [32].

These differences in metal content can be due to variations in the composition of soil type, sampling location, sample handling, and the presence of agents that increase or decrease metal content [33].

## 4. Conclusion

Eight samples of rice from the Jimma market were analyzed for the concentration of essential (Ca, K, Mg, and Fe) and nonessential (Cr, Ni, Cd, and Pb) metals. To prepare the clear solution sample, we used the wet digestion method using the reagents HNO_3_, H_2_O_2_, and HClO_4_. According to precision, accuracy, % RSD, and recovery, the optimized wet digestions for analysis were effective for all essential and nonessential metals. FAAS was used to analyze the essential (Ca, K, Mg, and Fe) and nonessential (Cr, Ni, Cd, and Pb) metals in this study. The concentration of nonessential metals (Ni, Pb, and Cd) exceeded the recommended limit by WHO/FAO. This may be due to various other factors such as production waste, fertilizer application, herbicide sprays, and various agricultural chemicals polluting the canal water, which are likely among the reasons for the high metal values of rice. This metal may have a hazardous effect on the health of the community. Therefore, care and responsibility must be taken to minimize the consumption of this toxic metal in diet. Proper maintenance of environmental protection laws is necessary to reduce pollution. Additionally, an in‐depth investigation should include all possible heavy metals missed in the current study due to certain constraints.

## 5. Limitations of the Study

The limitations of this study include the reliance on existing analytical methods without the development or optimization of novel techniques. While the employed methods are well‐established and suitable for elemental analysis, the study did not explore or incorporate advanced or emerging analytical techniques that could potentially offer higher sensitivity, accuracy, or efficiency. Consequently, the focus was primarily on assessing the elemental composition of rice samples using standard methods, which may limit the detection of trace levels of certain elements or the ability to analyze complex matrices more comprehensively. Future research could benefit from method development efforts to enhance analytical performance and broaden the scope of elemental detection.

## Funding

The authors express their gratitude for the financial support provided by Jimma University, Ethiopia.

## Conflicts of Interest

The authors declare no conflicts of interest.

## Supporting Information

The supporting information of this manuscript includes detailed notes for the sample preparation & digestion procedures, method validation techniques, and statistical analysis tools involved. Moreover, supporting data also includes sample site map (Figure S1), calibration curves (Figures S2–S8) for the various elements involved in these investigations, instrument working details, and working standard solutions involved in the study.

## Supporting information


**Supporting Information** Additional supporting information can be found online in the Supporting Information section.

## Data Availability

The data that support the findings of this study are available in the supporting information of this article.
