# Supplementary material for: Analysis of Essential and Nonessential Elements in Local and Imported Raw and Cooked Rice (Oryza sativa) Samples in Ethiopia
Source: Int J Anal Chem. 2026 Apr 28;2026:1237306. doi: 10.1155/ianc/1237306 (PMC13125719; doi:10.1155/ianc/1237306)
Supplement: Supplementary file 1 — Supporting Information Additional supporting information can be found online in the Supporting Information section. [file IANC-2026-1237306-s001.zip › TG-Revised SI-251114.docx]

***Supplementary Information***

**Analysis of Essential and Non-Essential Elements in Local and Imported Raw and Cooked Rice *(Oryza sativa****)* **Samples in Ethiopia.**

**Tamene Tadesse Beyene ^1,^ *, Tesfaye Gudina ^1^, Abebe Diro ^1^, Mulugeta Tesema Efa ^2^, Faisal Mukhtar ^3^**

1Department of Chemistry, College of Natural Sciences, Jimma University, Jimma, Oromia, Ethiopia,

2Department of Chemistry, Collage of Natural & Computational Science, Dambi Dollo University, Dambi Dollo, Oromia,Ethiopia,

3Institute of Physics, The Islamia University of Bahawalpur 63100, Pakistan

*Corresponding author

Beyene T.T. (Email: [tamene.tadesse@ju.edu.et](mailto:tamene.tadesse@ju.edu.et))

**Supportive Note 1**

1. **Preparation of solution**

***Preparation of Stock and Standard Solution***

Calibration curves were obtained for each of the metals by running a range of concentrations of the freshly prepared standard solutions in their respective linear ranges. The calibration curve was determined for the linear dynamic range from the standard serial solution concentration Vs instrument responses. The serially standardized solution was prepared by diluting 1000 ppm stock solution of (Ca, Mg, K, Fe, Ni, Cr, Cd, and Pb).

***Preparation of Metal Standard Mixture for Spiking***

A standard mixture solution containing all the metals of the study at different concentrations was prepared from stock solution (1000 pm) of each metal in a 50 mL volumetric flask and diluted with deionized water to the mark.

1. **Method Validation**

In the analysis of essential and non-essential metals in rice samples, reference materials, blanks and spikes were included for the analytical method validation. Blank analyses were carried out in order to check interference from instruments. Individual reference standards of metals (Ca, Mg, K, Fe, Ni, Cr, Cd and Pb) were used to identify and quantify the level of samples. Mean and standard deviation were calculated from positive quantifiable samples only. Evidence to prove this method was applicable for their intended purpose by determination of the analytical figure of merits. These figures of merits are Detection limit, Accuracy, Precision, Sensitivity

***Detection and Quantification Limit***

The method detection limit is the smallest mass of analysis that can be distinguished from statistical fluctuations in the blank, which usually corresponds to the standard deviation of the blank solution times a constant. The limit of detection is most commonly defined as the amount of analyte that gives a signal equal to three times the standard deviation of a blank. In this study, the reagent, which does not contain the sample (HNO_3_, HClO_4_ and H_2_O_2_), were digested. After digestion, a reading was taken for each blank and the standard deviation of these was calculated.

MDL = 3.3(SD) ….................................................................... (1)

The lowest concentration level at which a measurement is quantitatively meaningful is called the limit of quantification (MQL). The MQL is most often defined as 10 times the signal/noise ratio if the noise is approximated as the standard deviation of the blank; the MQL is 10 × SD of the blank. The MQL was calculated by multiplying the pooled standard deviation of the reagent blank by ten.

MQL =10(SD) ………………………………………………… (2)

***Precision and Accuracy***

The precision of an analytical procedure describes the closeness of individual measures of an analyte among a series of measurements. It is usually expressed as the variance (s^2^), standard deviation (SD), and relative standard deviation (RSD) of replicate measurements.

Precision and accuracy of the result were assessed by determining recovery and repeatability by spiking known concentrations of analyte and repeated measurements. In doing so, each sample was spiked in replicates of five at near mid-range calibration concentration. The spiked sample was digested and analyzed following the same analytical procedure as the rice sample. Precision is expressed as the relative standard deviation (RSD) of replicating results. The relative standard deviations of the samples were obtained as:

%RSD = $\frac{standarddevaition}{meanvaleu}$x100 ---------------------------------- (3)

Accuracy is how close the measured value is to the real or agreed to measure value. The accuracy of the optimized procedure was evaluated by spiking experiments. Accuracy was expressed as the percent recovery of an analyte that has been spiked to the samples in a known concentration before digestion and subsequent analysis.

The percentage recoveries of the analyte were calculated to evaluate the accuracy of the analytical procedure. Recovery was then calculated as:

%R**=**$\frac{Conc. in Spiked Sample-Conce.unspiked Sample}{Amount Added.}\boldsymbol{\times}100$------------------- (4)

The acceptable ranges of percentage recovery for the studied metals will be within 80 –120 % for metal analysis.

The values obtained for the relative standard deviations (% RSD) were ≤ 15 % for all the samples, showing the good precision of this method.

1. **Statistical analysis**

The analysis of variance ANOVA was used to examine the significance level of all parameters measured. The Least Significant Difference (LSD) test was used for means comparison. The level of significance for the t-test and means comparison was p<0.05.

**
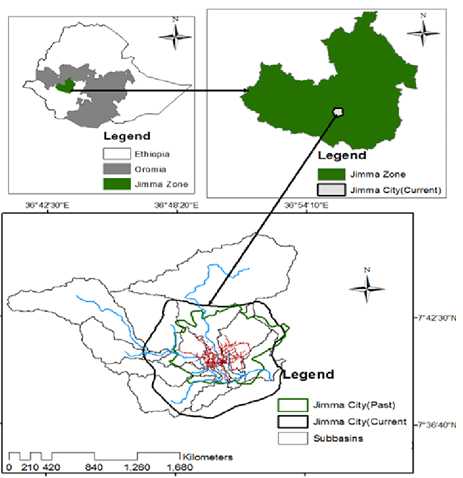
**

**Figure S1*:*** *Map of the study area sample collection site.*

**
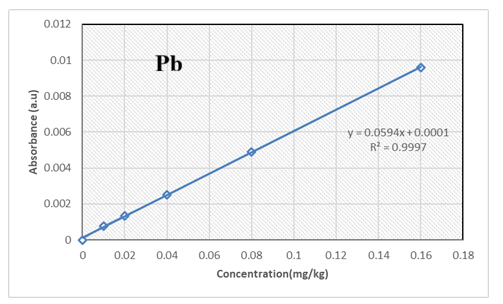
**

**Figure S2:** *Calibration curve of Pb determination*


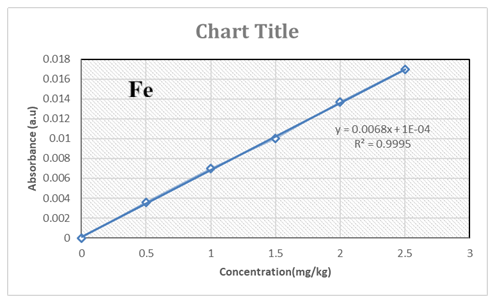


**Figure S3:** *Calibration curve of Fe determination*


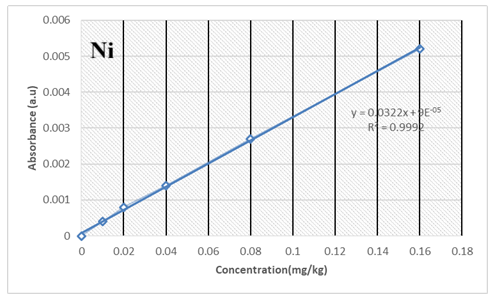


**Figure S4:** *Calibrations curve of Ni determination*


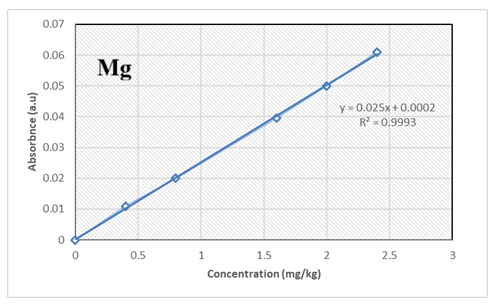


**Figure S5:** *Calibration curve of Mg determination*


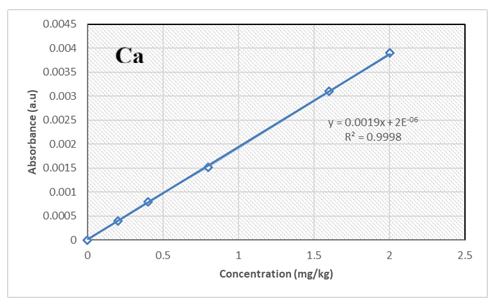


***Figure S6:*** Calibration curve of Ca *determination*

**
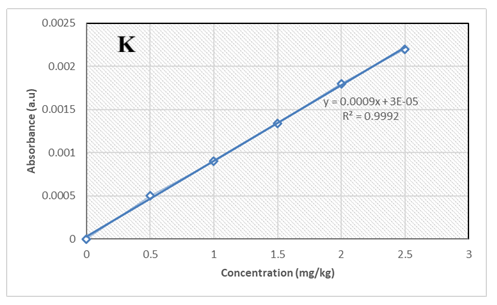
**

**Figure S7:** *Calibration curve of K determination*


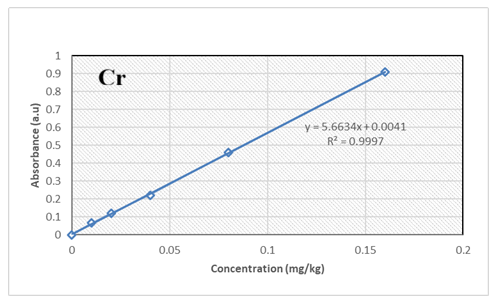


**Figure S8:** *Calibration curve of Cr determination*

**Table S1:** *A different procedure for digesting a powdered rice sample to determine the optimal method for 0.5 g of powdered rice.*

| **Procedure** | **Reagent** | **Temperature (^o^C)** | **Time(min)** | **Color** | **Remark** |
| --- | --- | --- | --- | --- | --- |
| A | 4 mL HNO_3_ 3 mL HClO_4_ | 180 | 180 | Yellow | Rejected |
| B | 4.5 mL HNO_3_ 1.5 mL HClO_4_ | 240 | 90 | Pale yellow | Rejected |
| C | 5.5 mL HNO_3_ 2 mL HClO_4_ | 180 | 190 | Bright yellow | Rejected |
| D | 5 mL HNO_4_ 2 mL HClO_4_ | 240 | 160 | Suspension yellow | Rejected |
| E | 2 mL HClO_4_ 3 mL H_2_O_2_ | 260 | 80 | colorless | Accepted |
| F | 3 mL HClO_4_ 3 mL HNO_3_ | 240 | 70 | Clear and colorless | Accepted |

**Table S2:** *Instrument working condition for FAAS (FS-210 FAAS)*

| **Property** | **Cr** | **Ni** | | **Cd** | **Pb** | **Fe** | | **Ca** | **Mg** |
| --- | --- | --- | --- | --- | --- | --- | --- | --- | --- |
| Wavelength (nm) | 357.9 | | 217.0 | 324.7 | 283.3 | | 248.3 | 279.5 | 285.2 |
| Slit width (nm) | 10 | | 7 | 7 | 10 | | 12 | 8 | 8 |
| Lamp | HCL | | HCL | HCL | HCL | | HCL | HCL | HCL |
| Lamp current (mA) | 2.0 | | 1.5 | 2.0 | 1.5 | | 2.0 | 2.0 | 2.0 |
| Gas | Acetylene | | Acetylene | Acetylene | Acetylene | | Acetylene | Acetylene | Acetylene |

**Table S3:** *Working standard solutions for calibration of FAAS instruments and calibration curve correlation coefficients.*

| **Metals** | **Intermediate solutions(ppm)** | **Concentration of calibration**  **standards solution (ppm)** | **Correlation Coefficient of determination(r^2^)** | **Calibration equation** |
| --- | --- | --- | --- | --- |
| Cr | 10 | 0.01,0.02,0.04,0.08,0.16 | 0.9997 | y =5.6634x +0.0041 |
| NI | 10 | 0.01,0.02,0.04,0.08,0.16 | 0.9992 | y = 0.0322x +9_E_^-05^ |
| Cd | 10 | 0.01,0.02,0.04,0.08,0.16 | 0.9994 | y = 0.0681x + 0.001 |
| Pb | 10 | 0.01,0.02,0.04,0.08,0.16 | 0.9997 | y = 0.0594x +0.0001 |
| Fe | 10 | 0.5,1.0,1.5,2.0,2.5 | 0.9992 | y = 0.0068x 1E-04 |
| Ca | 10 | 0.2,0.4,0.8,1.6,2 | 0.9998 | y = 0.0019x +2_E_^-06^ |
| K  Mg | 10  10 | 0.5,1,1.5,2,2.5  0.4,0.8,1.6,2,2.4 | 0.9992  0.9993 | Y=0.0009x +3_E_^-05^  y = 0.025x + 0.0002 |

Y=Absorbance and X=Concentration

**Table S4:** *IDL, MQL and MDL (mg/L) for Rice samples (n = 3 for MDL and MQL)*

| Metals | IDL(mg/Kg) | MDL(mg/Kg) | MQL(mg/Kg) |
| --- | --- | --- | --- |
| Cr | 0.0012 | 0.015 | 0.343 |
| Ni | 0.002 | 0.100 | 0.109 |
| Cd | 0.001 | 0.021 | 0.275 |
| Pb | 0.003 | 0.005 | 0.061 |
| Fe | 0.036 | 0.053 | 0.275 |
| Ca | 0.027 | 0.130 | 0.028 |
| K | 0.014 | 0.020 | 0.038 |
| Mg | 0.024 | 0.041 | 0.015 |
