# Supplementary figures and images for: Analysis of Essential and Nonessential Elements in Local and Imported Raw and Cooked Rice (Oryza sativa) Samples in Ethiopia
Source: Int J Anal Chem. 2026 Apr 28;2026:1237306. doi: 10.1155/ianc/1237306 (PMC13125719; doi:10.1155/ianc/1237306)

## Slide 1
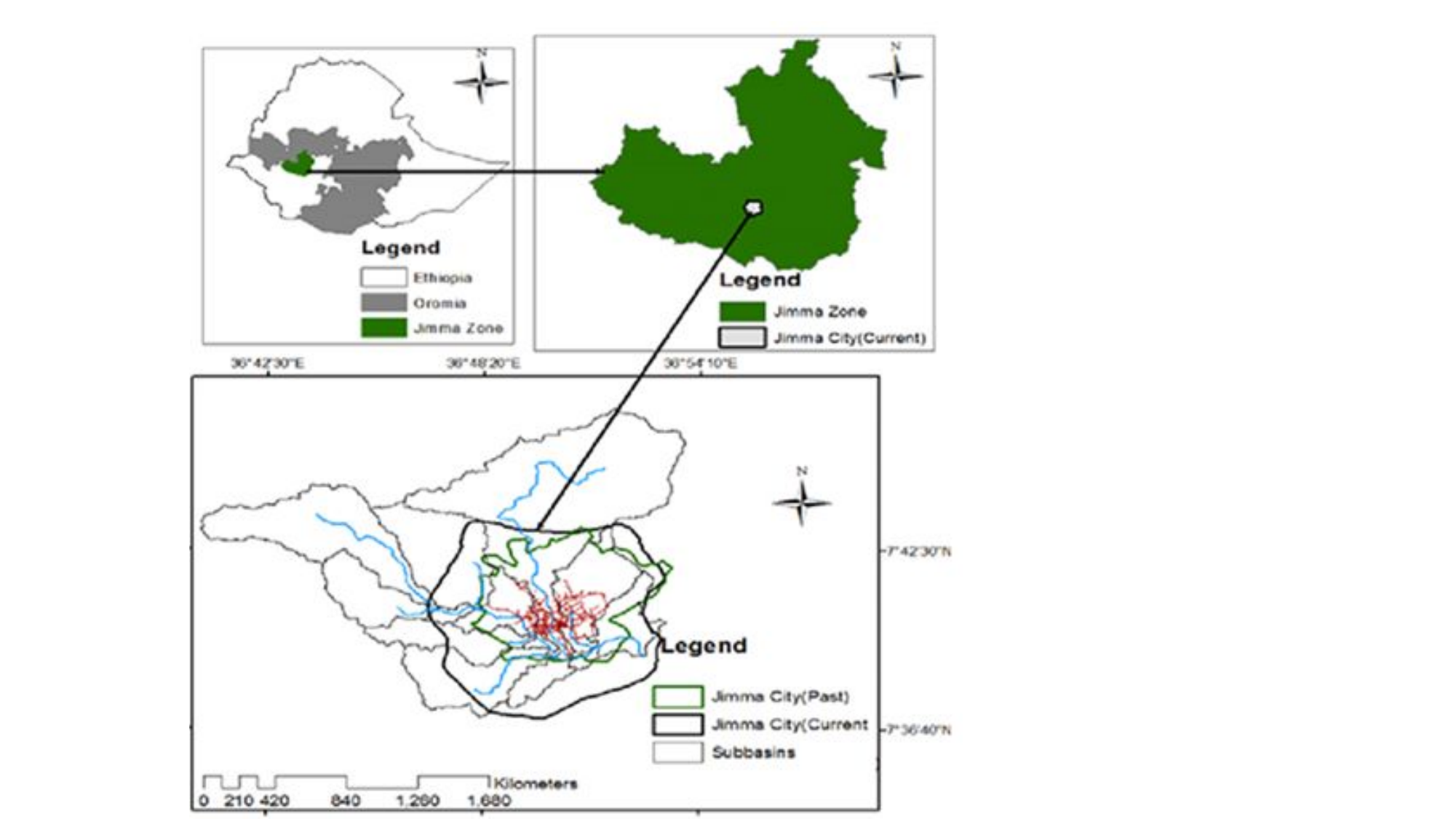

## Slide 2
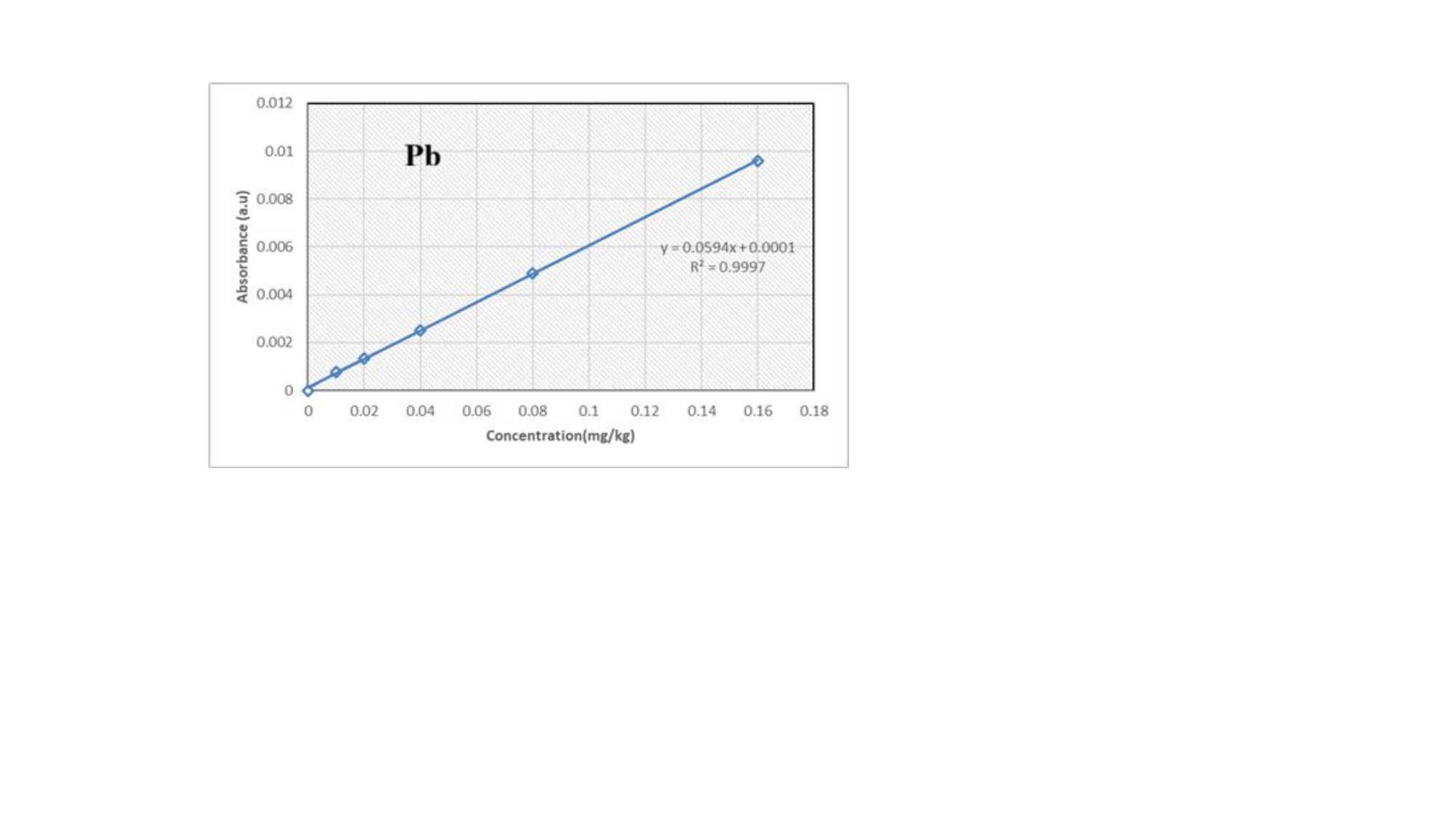

## Slide 3
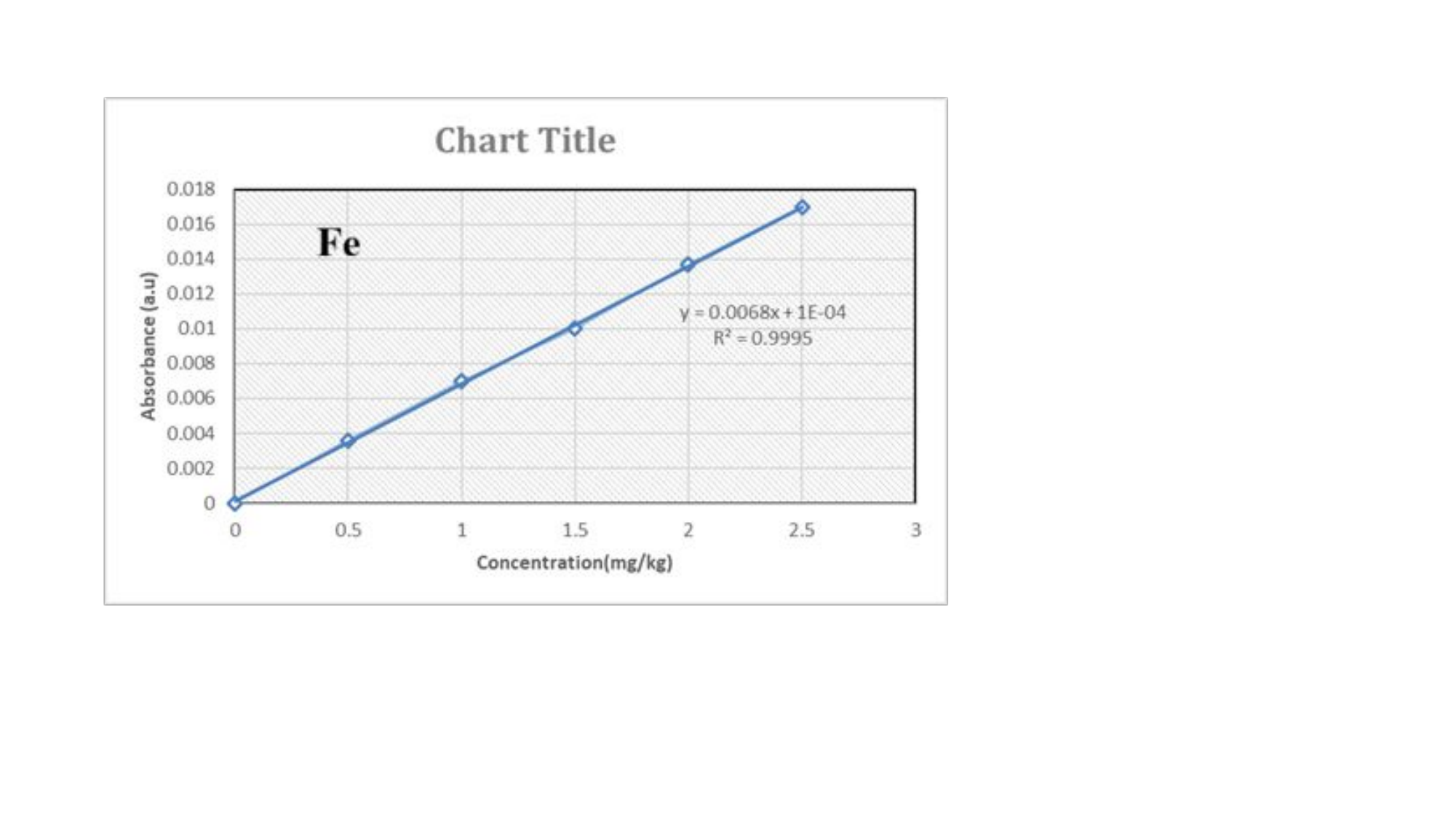

## Slide 4
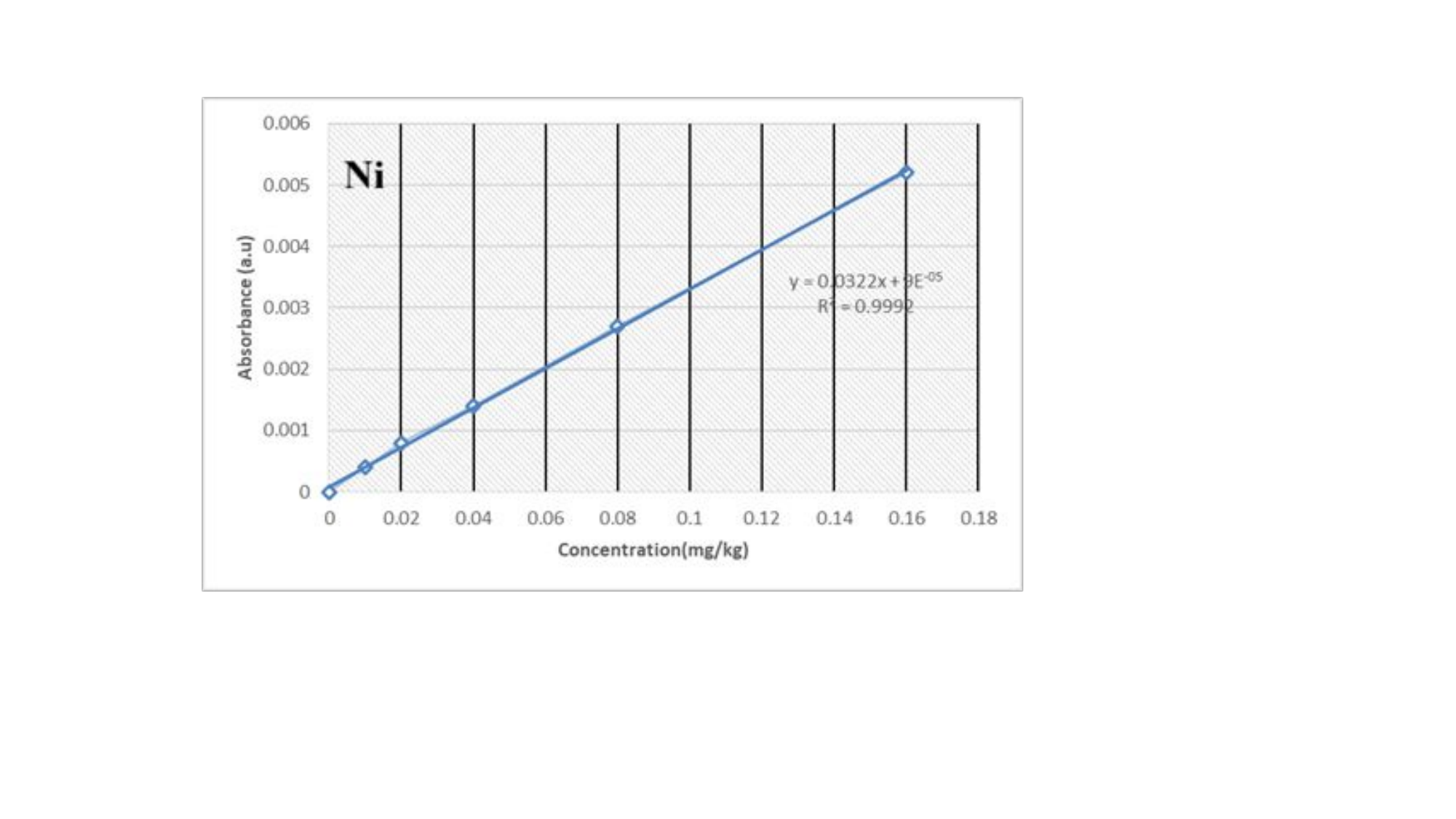

## Slide 5
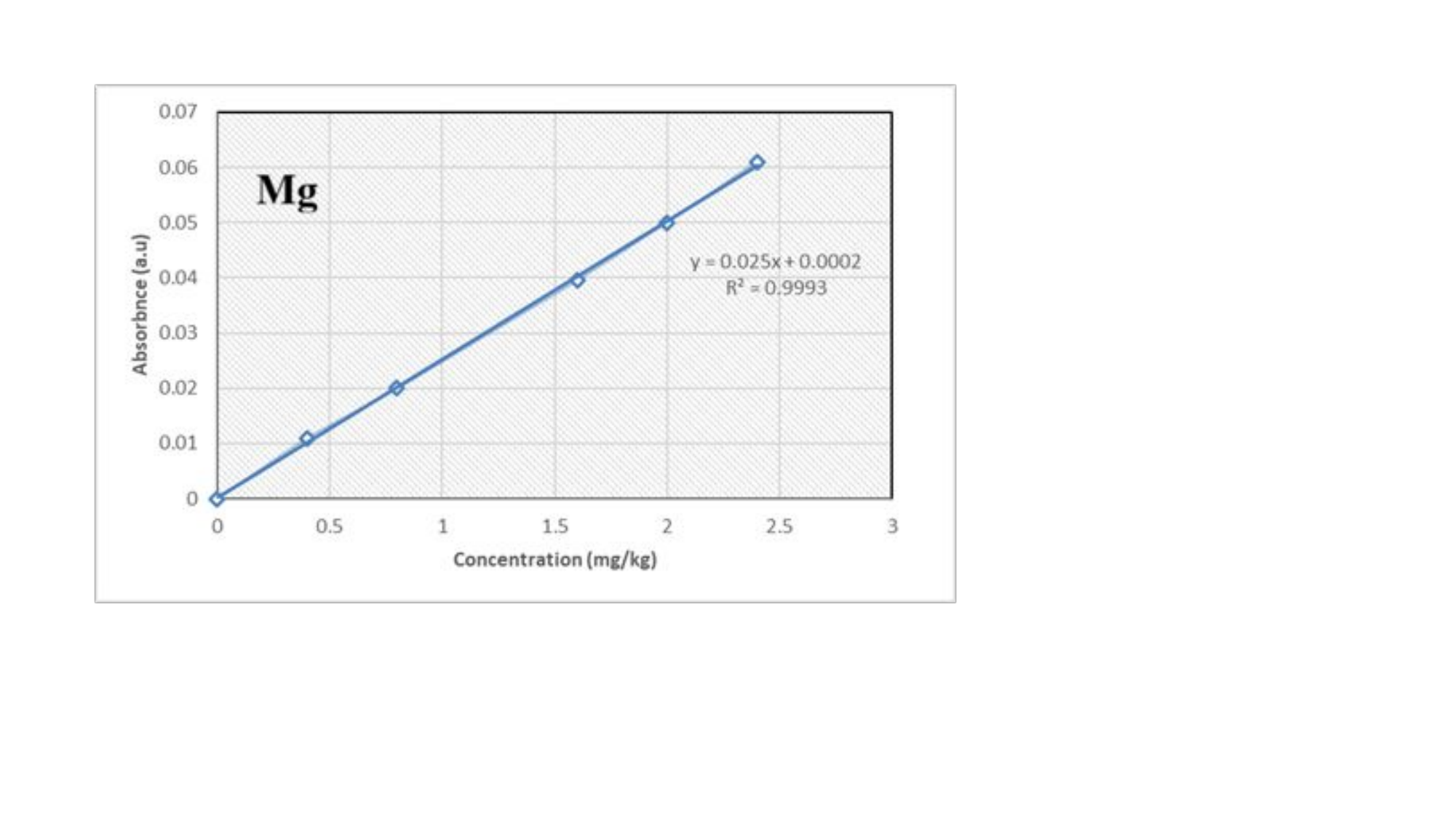

## Slide 6
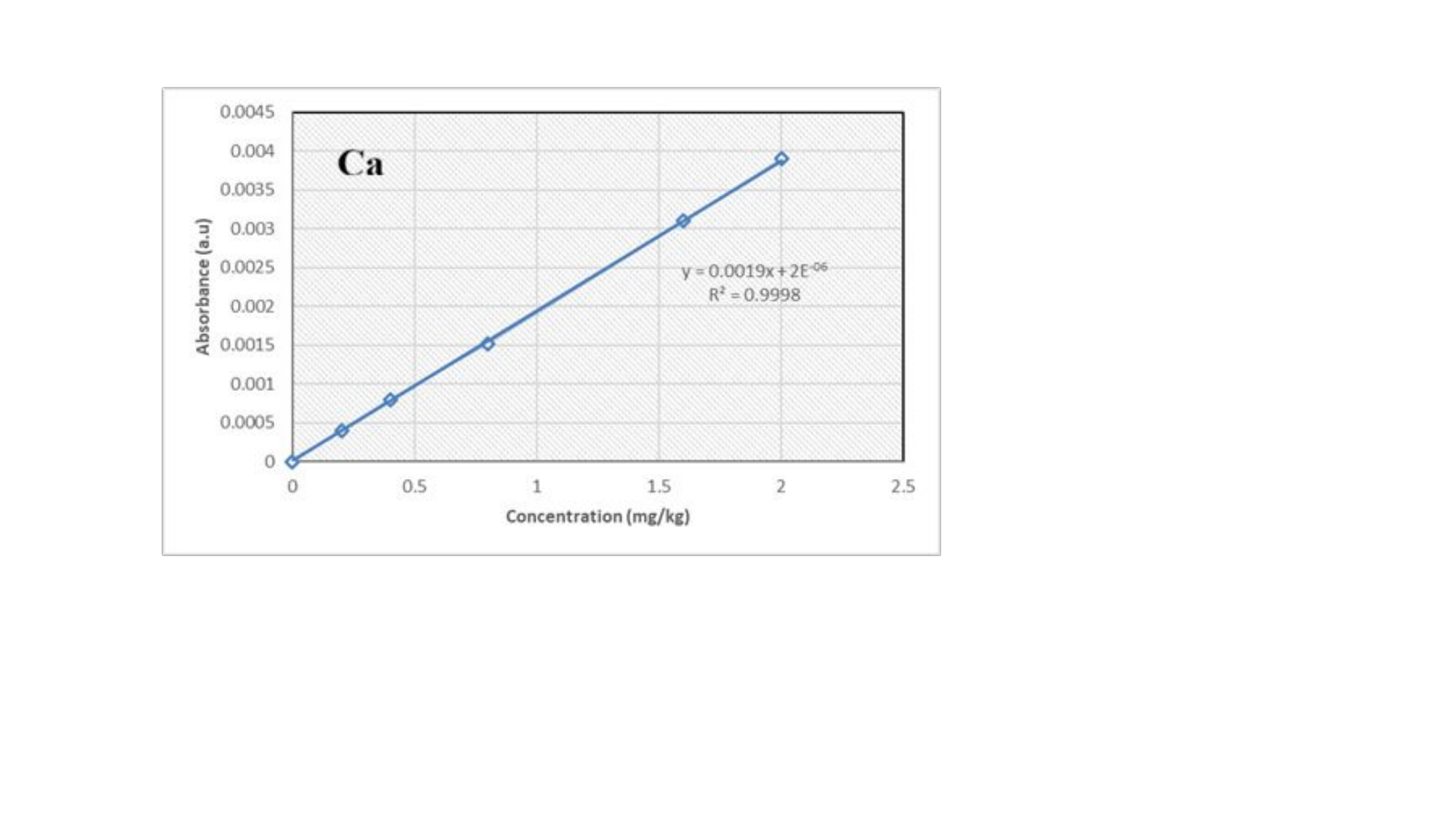

## Slide 7
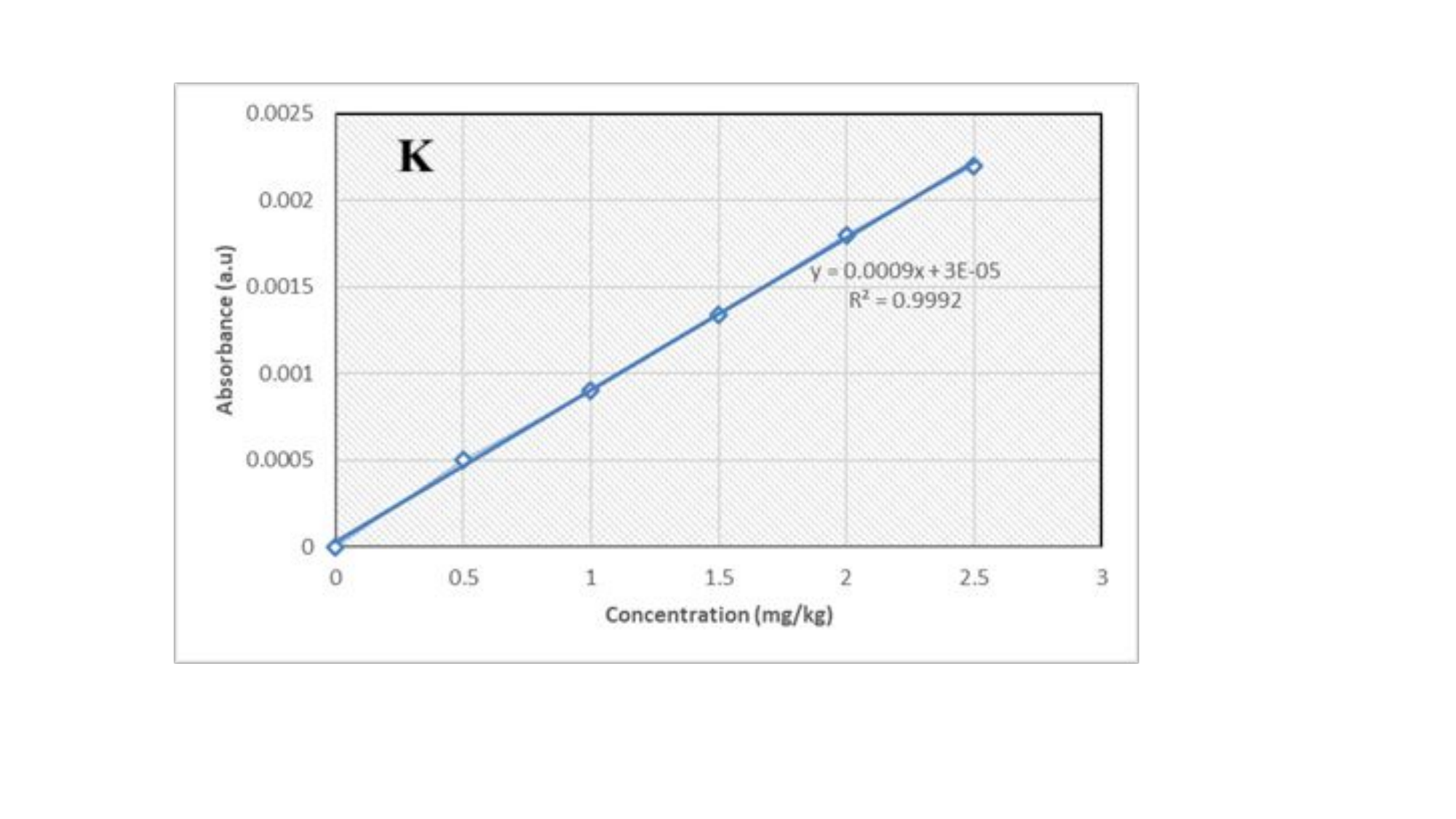

## Slide 8
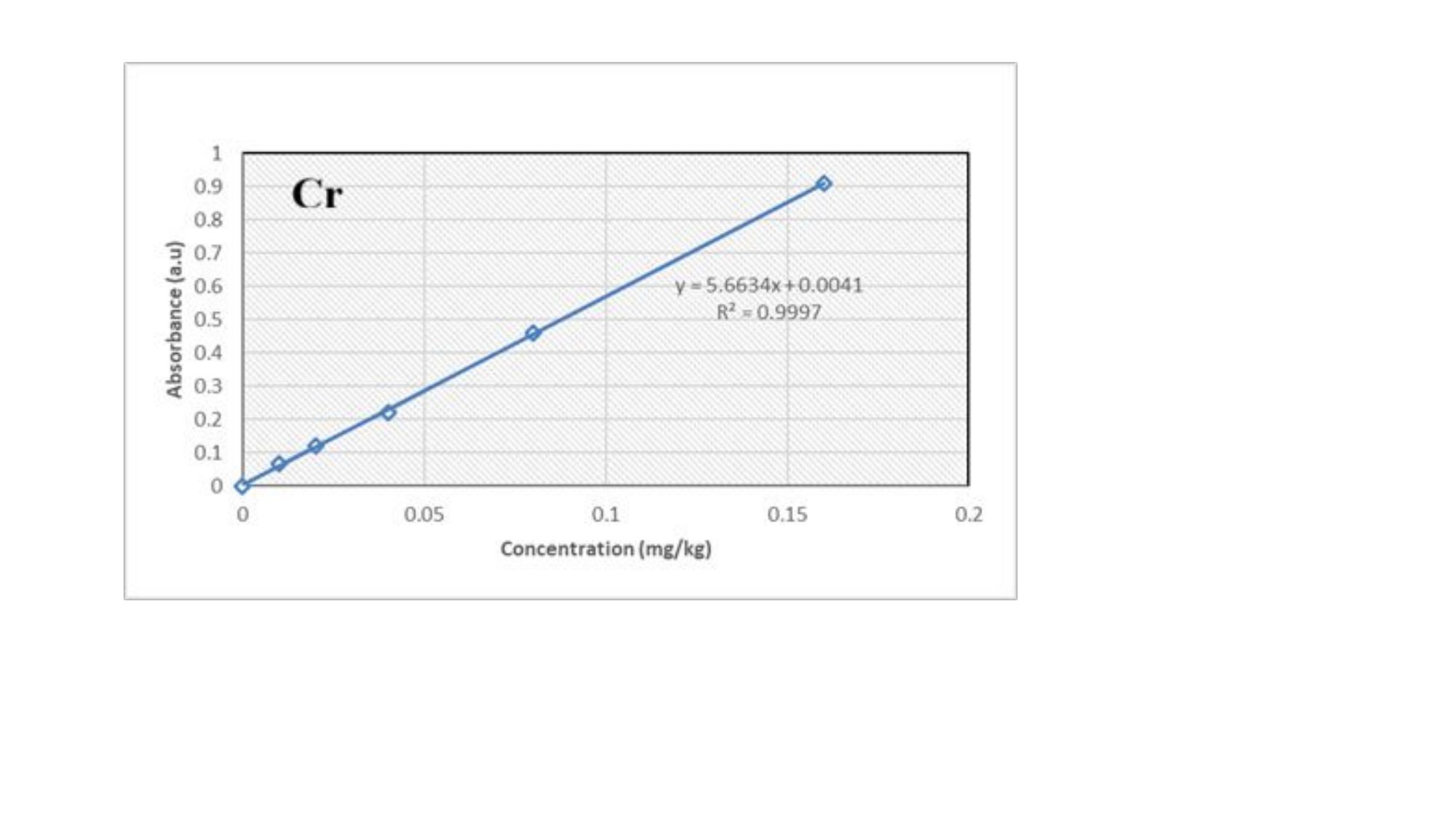

Supplement: Supplementary file 1 — Supporting Information Additional supporting information can be found online in the Supporting Information section. [file IANC-2026-1237306-s001.zip › Supplementary Figure.pptx]
